# Establishment of an antepartum predictive scoring model to identify candidates for vaginal birth after cesarean

**DOI:** 10.1186/s12884-020-03231-0

**Published:** 2020-10-20

**Authors:** Qiuping Liao, Jinying Luo, Lianghui Zheng, Qing Han, Zhaodong Liu, Wei Qi, Tingting Yang, Jianying Yan

**Affiliations:** Department of Gynecology and Obstetrics, Fujian Provincial Maternity and Children’s Hospital, Affiliated Hospital of Fujian Medical University, Daoshan Road 18, Gulou District, Fujian 350000 Fuzhou, China

**Keywords:** Cesarean delivery (CD), Trial of labor after cesarean (TOLAC), Vaginal birth after cesarean (VBAC), Factors, Prediction

## Abstract

**Background:**

Evidence-based medicine has shown that successful vaginal birth after cesarean (VBAC) is associated with fewer complications than an elective repeat cesarean. Although spontaneous vaginal births and reductions in cesarean delivery (CD) rates have been advocated, the risk factors for VBAC complications remain unclear and failed trials of labor (TOL) can lead to adverse pregnancy outcomes.

**Methods:**

To construct an antepartum predictive scoring model for VBAC. Retrospective analysis of charts from 1062 women who underwent TOL at no less than 28 gestational weeks with vertex singletons and no more than one prior CD.

**Results:**

We constructed our scoring model based on the following variables: maternal age, previous vaginal delivery, interdelivery interval (time between prior cesarean and the following delivery), presence of prior cesarean TOL, dystocia as prior CD indication, intertuberous diameter, maternal predelivery body mass index, gestational age at delivery, estimated fetal weight, and hypertensive disorders. Previous vaginal delivery was the most influential variable. The nomogram showed an area under the curve of 77.7% (95% confidence interval, 73.8–81.5%; sensitivity, 78%; specificity, 70%; cut-off, 13 points). The Kappa value to judge the consistency of the results between the predictive model and the actual results was 0.71(95% confidence interval 0.65–0.77) indicating strong consistency. We used the cut-off to divide the VBAC women into two groups according to the success of the TOL. The maternal and neonatal outcomes such as labor time, number of deliveries by midwives, postpartum hemorrhage, uterine rupture, neonatal asphyxia, puerperal infection were significantly different between the two groups.

**Conclusions:**

Our predictive scoring model incorporates easily ascertainable variables and can be used to personalize antepartum counselling for successful TOLs after cesareans.

## Background

According to World Health Organization (WHO) data, the rate of cesarean delivery (CD) increased an average of 46.2% between 2007 and 2008 in China, with some areas having increases > 60% [1]. China has one of the highest CD rates around the world.[1] A previous CD history has been considered an indication for cesarean due to fears of complications of vaginal birth after cesarean (VBAC). However, the evidence has shown that VBAC is associated with less maternal morbidity, fewer complications in future pregnancies, smaller overall cesarean delivery rates, and improved postnatal health-related quality of life at the population level than elective repeat cesareans [2–6]. Thus, spontaneous vaginal births and CD rate reductions have been advocated, along with prevention of VBAC complications [7] to improve pregnancy outcomes. This study evaluated the risks associated with trial of labor after cesarean (TOLAC) to establish a VBAC antepartum scoring model that can identify VBAC candidates among women with a previous low transverse CD.

## Methods

### Population

For this retrospective descriptive study, we extracted data from 1062 women with vertex singletons and no more than one prior CD, who had undergone TOL (at no less than 28 gestational weeks) at the Fujian Provincial Maternity of the Children’s Hospital in China between 2011 and 2016. We excluded data from women with abortions for abnormal fetal development and those who had undergone previous CDs for uterine malformations. We divided the data according to cases undergoing VBAC (n = 910) and cases who required CDs due to failed TOLs (*n* = 152), for a VBAC success rate of 85.69%. The ethics committee of the institute approved the conduct of the study (FMCH2018-127). The requirement for individual patient consent was waived as data were extracted from an anonymized database and our study did not influence patient care.

### Information collection

We recorded the following clinical information from patients’ charts: maternal age, pregnancy and prenatal body mass indexes (BMI), education degree, occupation, family, medical, and reproductive histories, previous cesarean delivery situation (cesarean indications, operation time, incision healing, uterine incision direction), pregnancy complications, uterine scar thickness, maternal pelvic dimensions, abdominal perimeter, uterine height, delivery gestational age, estimated fetal weight, labor process, number of deliveries by midwives, interdelivery interval, uterine rupture rate, postpartum hemorrhage rate, neonatal asphyxia rate, puerperal infection rate, hospital stay, and hospital expense. The interdelivery interval is the time between a prior cesarean and a following delivery. The estimated fetal weights were evaluated using a color Doppler ultrasound within 3 days before delivery.

### Methods

We expressed descriptive data as counts and percentages for qualitative variables and as means and SDs for continuous variables. We assessed successful VBAC indication factors by comparing qualitative variables using a Pearson *χ*^2^ or a Fisher exact test, and quantitative variables using a Student *t* or a *t’* test. The significantly different factors between groups were input into the multivariate logistic regression model. We considered statistical significance at *P* < 0.05. We used the variables associated with successful TOLAC after controlling for confounding factors to create our scoring model. We assigned each variable a score of 1 to 5, and calculated the likelihood of VBAC for the distribution of scores. We then applied the scoring model to all the individuals in the study population to calculate individual scores. Next, we assessed the predictive ability of the scoring model using an area under the receiver operating characteristic (ROC) curve, and calculated the optimal cut-off point accordingly. We divided the VBAC population into two groups based on the cut-off scores. Finally, we verified the safety and feasibility of the scoring model by comparing the pregnancy outcomes between the two groups. We analyzed the predictive scoring model to judge the consistency of the results between the predictive model and the actual results using Cohen’s Kappa coefficient analysis. We performed all statistical analyses using the SPSS 19.0 statistical software (IBM, Armonk, NY, USA).

## Results

Table 1 presents the demographic and obstetric characteristics of the study population. The women in the VBAC group were more likely to have greater mean intertuberous (IT) diameters and lower mean predelivery BMIs, fundal heights, maternal abdominal circumferences, gestational ages at delivery, and estimated fetal weights than those in the failed TOL group. The factors that increased the likelihood of successful TOL included maternal age at delivery < 35 years, interdelivery interval between 2 and 4 years, experience of TOL during prior CD, previous vaginal delivery, absence of dystocia during prior CD, and absence of hypertensive disorders.

**Table 1:** Population characteristics

Characteristic	VBAC (*n* = 910)	Failed TOL (*n* = 152)	*t*/*t*′ or *χ*^2^ value	*P* value
IT diameter (cm)	8.19 ± 2.69	7.95 ± 0.34	2.574	0.010
Maternal predelivery BMI (kg/m^2^)	25.68 ± 3.21	27.45 ± 3.13	−6.327	< 0.001
Fundal height (cm)	31.56 ± 3.50	33.39 ± 2.52	−7.805	< 0.001
Maternal AC (cm)	96.31 ± 9.59	99.26 ± 5.87	−3.673	< 0.001
Gestational age at delivery (weeks)	36.57 ± 3.57	38.42 ± 2.29	−8.416	< 0.001
Estimated fetal weight (g)	2752.67 ± 753.56	3198.74 ± 517.45	−7.025	< 0.001
Maternal age at delivery			6.292	0.012
< 35 years	759 (83.4)	114 (75.0)		
≥35 years	151 (16.6)	38 (25.0)		
Interdelivery interval			3.913	0.048
2–4 years	450 (49.5)	62 (40.8)		
< 2 or > 4 years	460 (50.5)	90 (59.2)		
TOL during prior CD			5.497	0.019
Yes	159 (17.5)	15 (9.9)		
No	751 (82.5)	137 (90.1)		
Dystocia as prior CD indication			30.590	< 0.001
Yes	137 (15.1)	51 (33.6)		
No	773 (84.9)	101 (66.4)		
Previous vaginal delivery			10.813	0.001
Yes	121 (13.3)	6 (3.9)		
No	789 (86.7)	146 (96.1)		
Hypertensive disorders			5.894	0.015
Yes	29 (3.2)	11 (7.2)		
No	881 (96.8)	141 (92.8)		

Values are presented as mean ± standard deviation or *n* (%)

*VBAC *vaginal birth after cesarean; *TOL* trial of labor; *IT* intertuberous; *BMI* body mass index (at delivery, kg/m^2^); *maternal AC* abdominal circumference; *CD* cesarean delivery

Table 2 presents the results of our multivariate logistic regression model used to evaluate the independent effect of all significant variables identified through the univariate analysis. All the variables were predictive of TOL success except for fundal height and maternal abdominal circumference. We found that a previous vaginal delivery was the most significant predictor of VBAC success (odds ratio [OR], 4.795; 95% confidence interval [CI], 4.877–12.248).

**Table 2:** Factors associated with vaginal birth after cesarean in multivariable logistic regression

Variable	OR	95% CI	*P* value
Maternal age at delivery	1.914	1.125–3.254	0.017
Without previous vaginal delivery	4.795	1.877–12.248	0.001
Interdelivery interval	1.622	1.005–2.618	0.048
Without TOL during prior CD	1.934	1.105–3.385	0.021
Dystocia as prior CD indication	2.849	1.943–4.177	< 0.001
IT diameter	0.163	0.079–0.334	< 0.001
Maternal predelivery BMI	1.130	1.046–1.221	0.002
Gestational age at delivery	1.233	1.148–1.323	< 0.001
Estimated fetal weight	1.001	1.000–1.001	0.031
Hypertensive disorders	3.472	1.212–9.944	0.020

*OR* odds ratio; *CI* confidence interval; *TOL* trial of labor; *CD* cesarean delivery; *IT* intertuberous; *BMI* body mass index (at delivery, kg/m^2^)

We graded independent factors based on the normal range of international pregnancy physiological indexes, and according to the clinical characteristics of uterine scar, pregnancy physiological changes, research data at home and abroad, and the ACOG VBAC guidelines. All the variables were scored based on the above gradings and we combined the OR values in the multivariate logistic regression model, as presented in Table 3.

**Table 3:** Predictive scoring model for deliveries before labor during vaginal birth after cesarean

Variable	Points	Variable	Points
Maternal age at delivery (years) <35 ≥ 35	2 0	IT diameter (cm) > 9.0 8.0–9.0 7.5 7.0 < 7.0	3 2 1 0 -1
Previous vaginal delivery yes no	4 0	Maternal pre-delivery BMI (Kg/m^2^) < 30 ≥ 30 and <50 ≥ 50	2 1 0
Interdelivery interval (years) 2–4 <2 or >4	2 0	Gestational age at delivery (weeks) <34 ≥ 34 and <37 37–40 ≥ 41	3 2 1 0
TOL during prior CD yes no	1 0	Estimated fetal weight (g) <1750 2000 ± 250 2500 ± 250 3000 ± 250 3500 ± 250 4000 ± 250 >4250	5 4 3 2 1 0 -1
Dystocia as prior CD indication no yes	1 -1	Hypertensive disorders no GH or CH Preeclampsia Severe Preeclampsia Eclampsia	3 2 1 0 -3

*VBAC* vaginal birth after cesarean; *IT* Intertuberous; *BMI* body mass index (at delivery, kg/m^2^); *GH* gestational hypertension; *CH* chronic hypertension

**Fig. 1:**
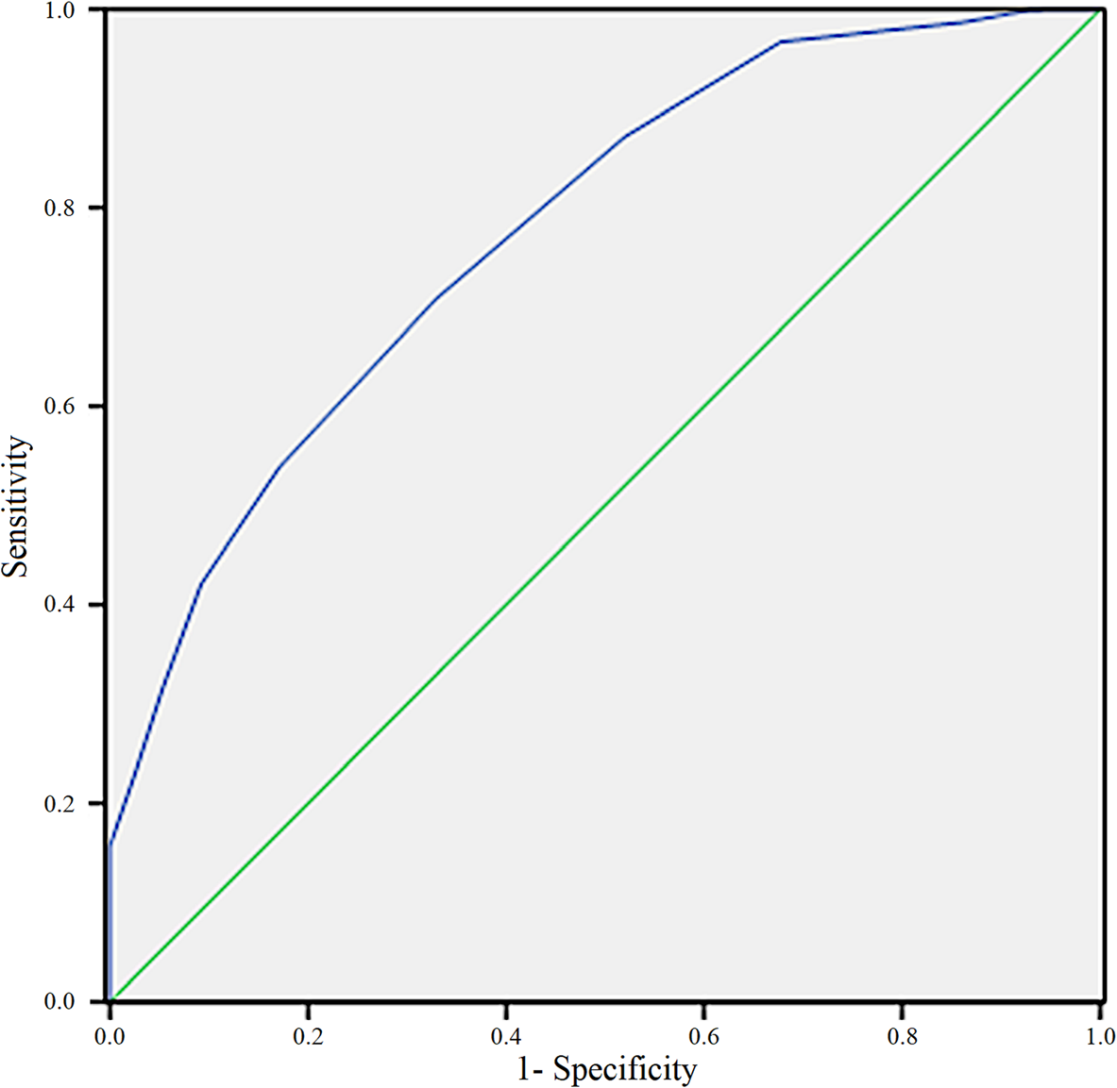
Receiver operating characteristic curve for the predictive scoring model for vaginal birth after cesarean

We scored each case by the sum of all the independent factors points. Figure 1 shows the corresponding ROC curve for VBAC prediction with an area under the curve of 0.777 (95% CI, 0.738–0.815). The optimal cut-off point score was 13 in the ROC curve (sensitivity, 78%; specificity, 70%). Thus, patients with scores ≥13 points had a high chance of undergoing VBAC (Fig. 1).

The Kappa value to judge the consistency of the results between the predictive model and the actual results was 0.71(95% confidence interval 0.65–0.77) indicating strong consistency (Table 4).

**Table 4:** Consistency of the results between the predictive model and actual clinically observed TOL results

Predictive TOL results of the model	Clinical actual TOL results		Total	Kappa coefficient	Kappa coefficient 95% confidence interval
	VBAC	Failed TOL			
VBAC (total score ≥ 13)	854	26	880	0.709	0.650–0.768
Failed TOL (total score < 13)	56	126	182		
Total	910	152	1062		

*VBAC* vaginal birth after cesarean; *TOL* trials of labor

Within the VBAC population, the women with scores ≥13 had less mean labor time, and lower rates of deliveries by midwives, postpartum hemorrhage, uterine rupture, neonatal asphyxia, and puerperal infection than women with scores < 13, as presented in Table 5.

**Table 5:** Descriptive characteristics of pregnancy in cases of vaginal birth after cesarean

Variable	≥13 Points (*n* = 854)	< 13 Points (*n* = 56)	*t* or *χ*^*2*^ value	*P* value
Mean labor time (h)	5.96 ± 4.47	6.66 ± 3.45	2.271	0.023
Midwifery rate	13 (1.52)	16 (28.57)	116.017	0.000
PPH rate	22 (2.58)	18 (32.14)	102.401	0.000
Uterine rupture rate	0	3 (5.36)	31.045	0.000
Neonatal asphyxia rate	6 (0.70)	7 (12.50)	43.903	0.000
Puerperal infection rate	7 (0.82)	8 (14.29)	50.770	0.000

Values are presented as mean ± standard deviation or *n* (%)

*PPH* postpartum hemorrhage

## Discussion

Successful VBAC has been associated with fewer major complications than repeat elective cesarean [8]. To improve the success rate of TOLAC, researchers worldwide have tried to elucidate the factors associated with successful VBAC and many models that predict the success of VBAC have been published [19–22]. However, these models have not been tested in the Chinese population, which differs in terms of ethnicity, culture, medical level, and medical laws (which prohibit the determination of fetal sex before birth). Hence, we designed this study to develop a new model by combining and improving approaches of existing models to predict successful intended VBAC reliably in the Chinese population.

In our study population, the mean maternal IT diameter and predelivery BMI were significantly different between the VBAC and failed TOL groups (*P* < 0.05). Also, advanced maternal age (≥35 years) was also confirmed to have an impact on the success of TOLAC. A couple of studies have similarly indicated that maternal age and BMI are the main factors associated with the success of VBAC [9, 10]. Investigators have also shown that maternal BMIs > 30 kg/m^2^ decrease the likelihood of VBAC, and that maternal BMIs > 50 kg/m^2^ reach failure VBAC rate of up to 87% [11]. The same authors also suggested that women with high BMIs should choose a repeat cesarean before labor in consideration of the difficulty in performing emergency cesareans [11]. In terms of the IT diameter, another investigation observed that a high IT diameter is significantly associated with increased likelihood of VBAC [12].

As for the medical history, various characteristics are predictive of VBAC success. For instance, a previous vaginal delivery has been associated with a high rate of successful delivery [10]. Ashwal et al. demonstrated that VBAC prediction is difficult in women without previous vaginal deliveries [13]. The 2015 Queensland Clinical Guidelines state that previous vaginal birth, whether before or after a cesarean, is a strong predictor of successful TOLAC [14]. We also found that previous vaginal delivery is the most significant variable in the multivariate logistic regression analysis (OR, 4.795). Similar to the results of other studies, TOL before a prior CD increases the likelihood of VBAC except in the cases with dystocia as a CD indication. The stagnation of the labor process and the failure of the fetal head to descend decrease the likelihood of VBAC [15]. In 2010, the American College of Obstetricians and Gynecologists also stated that one of the predictors of successful VBAC was the absence of dystocia as a prior CD indication [8]. As for the optimal interval of a subsequent pregnancy after CD to minimize adverse outcomes of pregnancy, most investigations have shown that 2–3 years after the prior CD seem appropriate, considering the healing of uterine scar (prolonged intervals change the scar toughness and may lead to uterine ruptures). Rietveld et al. showed that an interpregnancy interval < 24 months was not associated with decreased VBAC success and that success rates decreased when the interval increased [16]. Bujold and Gauthier found that an interdelivery interval < 18 months presented a higher risk factor of uterine rupture than intervals of 18–24 and > 24 months [17]. García-Benítez et al. also considered that an interdelivery interval > 18 months is safe [18]. Thus, the evidence suggests that an interval between 2 and 4 years increases the likelihood of successful VBAC.

Gestational age and birth weight have been also analyzed by researchers. Schmitz found that fetal macrosomia (birth weight > 4000 g) may increase the risk of failed TOLAC, and especially for women with no prior vaginal delivery, macrosomia decreases the rate of VBAC by 40% and increases the risk of uterine ruptures threefold [11]. The 2015 Queensland Clinical Guidelines state that birth weight < 4000 g and spontaneous onset of labor at less than 41 weeks of gestation increase the likelihood of VBAC [14]. TOLAC should also be considered for premature deliveries, whereas delayed pregnancies decrease the likelihood of VBAC. On detailed examination of our data, it can be seen that fundal heights were slightly lower in our population as compared to international standards. However, it is known that fundal height are influenced by ethnicity, with lower values seen in Asian populations [19]. Secondly, even after exclusion of women under 28 weeks of gestation, the mean gestational age of our sample was slightly on the lower side. This may have resulted in higher success of VBAC in our study as compared to published literature [5, 9]. Correspondingly, we also found that gestational age and birth weight were factors associated with successful VBAC. After analyzing the characteristics of pregnancy complications, we found that hypertensive disorders during pregnancy negatively influenced the success of VBAC.

Models based on factors associated with successful TOLAC can identify women likely to deliver vaginally after cesareans. In 2007, Grobman et al. presented a model to provide personalized TOLAC counseling for women early in the prenatal course (during their first examination), which included demographic, obstetric, and medical history variables [20]. The model is limited to certain populations with term pregnancies and clinical data on third trimester pregnancies (like complications and estimated fetal weights) are lacking, which decrease its prediction performance. Thus, we aimed at improving the model, and chose a population with both term and premature pregnancies and included clinical data during the whole pregnancy. A prediction model by Schoorel et al. presented in a multicenter retrospective study of 515 women with one previous cesarean undergoing TOL (cephalic presentation at term singleton pregnancy) had a small sample size and was limited by gestational age [21]. On the other hand, our model was based on a relatively large sample and the women were at ≥28 weeks of gestation. Hashima and Guise developed a prediction model that included the variable of fetal sex [22]. However, determining fetal sex before delivery goes against the medical law in China. Thus, the model is not suitable in China. The model by Smith et al. has the same problem; and is only suitable for women with ≥40 weeks of gestation. [23]. Thus, all of the available models present regional limitations and may not be appropriate in China. Our model presents some advantages: the population was relatively large, and the prediction area under the curve reached 0.777, under suitable conditions for China. Moreover, adding the points of the variables of our scoring model is convenient in the clinical practice for VBAC counseling.

## Conclusions

In our study, we generated a ROC curve for our VBAC predictive scoring model and set the cut-off point score at 13. The VBAC success rate in our study population was 92.8% in women who scored ≥13 points, indicating a relatively high prediction capability for our scoring model. The Kappa value to judge the consistency of the results between the predictive model and the actual results was 0.71(95% confidence interval 0.65–0.77) indicating strong consistency. We also found that the rates of adverse pregnancy outcomes in our VBAC population were lower in women with scores ≥13 than in those with scores < 13, indicating the relatively high safety of our model for clinical applications.

We are aware of the limitations of our study. Our model is based only on antepartum factors and did not include intrapartum variables such as cervical examination. The population was limited to one of the numerous hospitals in China, which has uneven medical levels. Therefore, our results should be interpreted with caution. However, we developed an appropriate Chinese population–based prediction model to counsel women on the mode of birth after cesareans. Our model can be useful while we wait for multi-center studies that result in standard recommendations on the mode of delivery after cesareans given the increasing CD rates and numbers of women with a history of CD.

## Data Availability

The datasets used and/or analyzed during the current study are available from the corresponding author on reasonable request.

## Abbreviations

VBAC: Vaginal birth after cesarean
CD: Cesarean delivery
TOL: Trials of labor
TOLAC: Trial of labor after cesarean
WHO: World Health Organization
BMI: Body mass indexes
ROC: Receiver operating characteristic
IT: Intertuberous
OR: Odds ratio
CI: Confidence interval
